# Vagus nerve stimulation for the therapy of Dravet syndrome: a systematic review and meta-analysis

**DOI:** 10.3389/fneur.2024.1402989

**Published:** 2024-07-09

**Authors:** Shuang Chen, Man Li, Ming Huang

**Affiliations:** ^1^Department of Neurology, Hubei Provincial Hospital of Integrated Chinese and Western Medicine, Hubei University of Chinese Medicine, Wuhan, China; ^2^Department of Neurology, Union Hospital, Tongji Medical College, Huazhong University of Science and Technology, Wuhan, China

**Keywords:** Dravet syndrome, vagus nerve stimulation, epilepsy, meta-analysis, systematic review

## Abstract

**Objective:**

Dravet syndrome (DS) is a refractory developmental and epileptic encephalopathy characterized by seizures, developmental delay and cognitive impairment with a variety of comorbidities, including autism-like behavior, speech dysfunction, and ataxia. Vagus nerve stimulation (VNS) is one of the common therapies for DS. Here, we aim to perform a meta-analysis and systematic review of the efficacy of VNS in DS patients.

**Methods:**

We systematically searched four databases (PubMed, Embase, Cochrane and CNKI) to identify potentially eligible studies from their inception to January 2024. These studies provided the effective rate of VNS in treating patients with DS. The proportions of DS patients achieving ≥50% reduction of seizure frequency were extracted from these studies. Meta-analyses were performed to respectively evaluate the efficacy of VNS for DS after 3, 6, 12, 18, 24 and 36 months.

**Results:**

Sixteen trials with a total of 173 patients were included. Meta-analyses showed that the pooled efficiency was 0.54 (95% CI 0.43–0.65) in the DS patients treated with VNS (*p* < 0.05). Meanwhile, the pooled efficiency respectively was 0.42 (95% CI 0.25–0.61), 0.54 (95% CI 0.39–0.69), 0.51 (95% CI 0.39–0.66), and 0.49 (95% CI 0.36–0.63) in the DS patients treated with VNS after 3, 6, 12 and 24 months (*p* < 0.05).

**Conclusion:**

This study suggests that VNS is effective in the treatment of DS. However, few studies have focused on VNS for DS, and there is a lack of high-quality evidence. Thus, high-quality randomized controlled trials are needed to confirm the efficacy of VNS in DS.

## 1 Introduction

Dravet syndrome (DS), also known as severe myoclonic epilepsy in infancy (SMEI), is a refractory developmental and epileptic encephalopathy characterized by seizures, cognitive impairment and developmental delay with multiple comorbidities, including speech dysfunction, ataxia, and autism-like behavior (1, 2). Meanwhile, DS as a severe congenital developmental genetic epilepsy, is associated with mutations in the gene of *SCN1A*, which encodes the voltage-gated sodium channel (VGSC) subunit Nav1.1 (3, 4). Previous studies have found that at least 80% cases of DS are linked to mutations in genes which encode VGSC subunits (*SCN1A* and *SCN1B*), which encode the Nav1.1α subunit and VGSC β1 subunit, respectively (5). Despite the clinical evidences have suggested effective therapies, including topiramate, valproic acid (VPA), cannabidiol, clobazam, fenfluramine and ketogenic diet, DS is known to be treatment-resistant (6). Conventional antiseizure medications (ASMs) often fail to control seizures of DS well. Relevant studies have shown that 45% of DS patients still have at least 4 tonic-clonic seizures each month after treated with at least 3 ASMs (7). Although the abnormal discharge of neurons is a leading pathophysiological manifestation of epilepsy including DS, owing to its complex and changeable property, the mechanism of epilepsy is yet unclear, which renders difficulty in the treatment of DS.

Vagus nerve stimulation (VNS), as a palliative therapy that stimulates the vagus nerves, is the main adjunct therapy to drug-resistant epilepsy (DRE) and provide an alternative for patients who are not candidates for respective surgery (8). Currently, VNS is the first neuromodulation device approved for the treatment of epilepsy and has been proven to be a safe and effective treatment for DRE (9). VNS was approved in the United States in 1997. As of 2020, VNS has been implemented in approximately 125,000 patients worldwide and is being used in more than 35,000 pediatric patients. It has also been approved as a long-term treatment for DRE in children (8). VNS, a non-pharmacological intervention that enhances seizure control, has also been employed in the treatment of patients with DS (9, 10). Studies have shown that VNS appears to be beneficial for children with *SCN1A* gene abnormalities associated with refractory epilepsy (11). Although the VNS is considered a viable treatment option for patients with DS, tuberous sclerosis complex (TSC) or Rett syndrome, the effectiveness of VNS for DRE with other rare gene mutations remains uncertain (10). Relevant studies have demonstrated that VNS therapy is both safe and effective in focal, generalized and combined types of epilepsy. VNS serves as a viable alternative treatment option for individuals with epilepsy who are not suitable candidates for surgery (12). In the DRE, a meta-analysis have showed that seizure reduction for the VNS devices were VNS 32.9, 44.4, and 53.5% at one, two and three years, respectively (13). Meanwhile, in another meta-analysis regarding DRE, VNS demonstrated the ability to significantly decrease seizures and improve quality of life in patients with TSC, while the reduced seizures were comparatively less pronounced in patients with DS (9). However, these studies did not discuss the efficacy of VNS in the treatment of DS at various durations. Recent studies about the effects of VNS on DS indicated that a more than 50% reduction in seizure frequency was observed in 36.4% (8/22), 54.5% (12/22), and 63.2% (12/19) of the patients at 12, 24, and 36 months, respectively (6). In the context of DS, research has demonstrated that VNS has the potential to decrease the frequency or duration of seizures by 50 to 75% in approximately 50% of children with DS (14). Meanwhile, a previous meta-analysis about DS has also found that adjunctive VNS to elicit a 50% seizure frequency reduction in 52.9% of patients with DS with a median seizure frequency reduction of 55% (15). However, this meta-analysis did not address the effectiveness of VNS in the treatment of DS at different various follow-up times. In a word, VNS has been found to be effective in managing DRE and could potentially serve as a safe and effective treatment option for DS. In children with DS, the role of VNS is not well described, and most of the studies have had a rather short follow-up period. Therefore, the efficacy of VNS to treat DS needs further verification. The objective of this study was to conduct a systematic literature review and perform a meta-analysis to evaluate the efficacy of VNS in pediatric patients with DS. Meanwhile, our study will also further explore the effectiveness of VNS after 3, 6, 12, 18, 24 and 36 months in the treatment of DS through meta-analysis.

## 2 Methods

### 2.1 Search strategy

Electronic databases (PubMed, Embase, Cochrane and CNKI) were searched from inception to January 2024. We used the following keywords: Dravet syndrome (DS) OR Dravet’s syndrome OR Dravets syndrome OR severe infantile myoclonic epilepsy OR severe myoclonic epilepsy of infancy OR severe myoclonic epilepsy in infancy (SMEI), AND vagus nerve stimulation (VNS) OR vagal activity OR vagal nerve stimulation OR vagal stimulation OR vagus stimulation. We also searched international trial registers, such as ClinicalTrials.gov, and screened the bibliographies of relevant reports. There were no date limitations or language restrictions. Reference lists of publications were manually searched.

### 2.2 Eligibility criteria

1. ≤ 18 years of age at the time of VNS device implantation.
2. All patients were implanted with a VNS therapy device for DS and underwent VNS surgery was performed in a standard manner under general anesthesia.
3. Response was defined as ≥50% reduction in baseline seizure frequency.

### 2.3 Study selection

Prior to comparing the selected articles, the authors conducted a comprehensive review of the full-text articles. They independently excluded non-relevant articles that were not relevant, such as review, case, graduation thesis, note, conference abstract and comment. The selected articles were then subjected to eligibility criteria to finalize the selection. Journal of biomedical Informatics (JBI) was used to evaluate the quality of the included references (Table 1).

**Table 1:** Quality evaluation of the included references.

Study	1	2	3	4	5	6	7	8	9	10	References
Nelia Zamponi	N	Y	Y	Y	N	Y	Y	Y	Y	Y	(16)
Ricardo O. Cersósimo (1)	Y	Y	Y	Y	N	Y	Y	Y	N	Y	(17)
Ricardo O. Cersósimo (2)	Y	Y	Y	Y	N	Y	Y	Y	N	Y	(18)
Servicio de Neurología	N	Y	Y	Y	N	Y	Y	Y	Y	Y	(19)
Iren Orosz	Y	Y	Y	Y	Y	Y	Y	Y	Y	Y	(20)
Anastasia Dressler	N	Y	Y	Y	N	Y	Y	Y	N	Y	(21)
Deepa Sirsi	Y	Y	Y	Y	N	Y	Y	Y	Y	Y	(12)
Brian J. Dlouhy	Y	Y	Y	Y	N	Y	N	Y	Y	Y	(22)
Stephen P. Fulton	N	Y	Y	Y	N	Y	Y	Y	N	Y	(11)
Rushna Ali	N	Y	Y	Y	N	Y	Y	Y	N	Y	(23)
Yijie Li	Y	Y	Y	Y	N	Y	Y	Y	Y	Y	(24)
Tong Zhang	Y	Y	Y	Y	N	Y	Y	Y	Y	Y	(25)
ZhiJi Wang	Y	Y	Y	Y	N	Y	Y	Y	Y	Y	(26)
Song Ee Youn	Y	Y	Y	Y	N	Y	Y	Y	Y	Y	(27)
Cem Boluk	Y	Y	Y	Y	N	Y	Y	Y	N	Y	(12)
Han Xie	Y	Y	Y	Y	N	Y	Y	Y	Y	Y	(10)

(1) Were there clear criteria for inclusion in the case series?; (2) Was the condition measured in a standard. Reliable way for all participants included in the case series?; (3) Were valid methods used for identification of the condition for all participants included in the case series?; (4) Did the case series have consecutive inclusion of participants?; (5) Did the case series have complete inclusion of participants?; (6) Was there clear reporting of the demographics of the participants in the study?; (7) Was there clear reporting of clinical information of the participants?; (8) Were the outcomes or follow up results of cases clearly reported?; (9) Was there clear reporting of the presenting site(s)/clinic(s) demographic information?; (10) Was statistical analysis appropriate? Y: Yes: N: No.

### 2.4 Data extraction and analysis

For all the articles in the study, we collected the following available data: first author’s name the year of publication, the location of study, the follow-up time, the number of DS patients with DS who received VNS treatment at baseline and the number of DS patients who became seizure-free or showed ≥50% reduction of seizure frequency. Meanwhile we also focused on extracting the data including adverse events, further patient outcome, study limitations. Descriptive statistics for the fraction of patients experiencing 50% reduction were computed for all 16 studies. For the studies under different treatment time (3, 6, 12, 18, 24 and 36 months), descriptive statistics was performed once for all data. Primary outcomes included seizure freedom rate and ≥50% seizure reduction rate, which was also defined as the responder rate. These outcomes were reported for large cohorts or extrapolated from smaller studies. The ≥50% seizure reduction rate was calculated by pooling the individual participant responses, when available and reported in each study. The effectiveness of VNS treatment for DS under different treatment time (3, 6, 12, 18, 24 and 36 months) was further compared.

### 2.5 Statistical analysis

Random-effects meta-analysis was performed in this study. The *I*^2^ statistic was used to estimate heterogeneity, and Egger’s test was used to estimate publication bias. Pooled proportions and exact binomial confidence intervals were reported. A *p*-value <0.05 was considered significant. Analysis was done by using Stata version 16.1 (StataCorp).

## 3 Results

### 3.1 Results of the search

**Figure 1:**
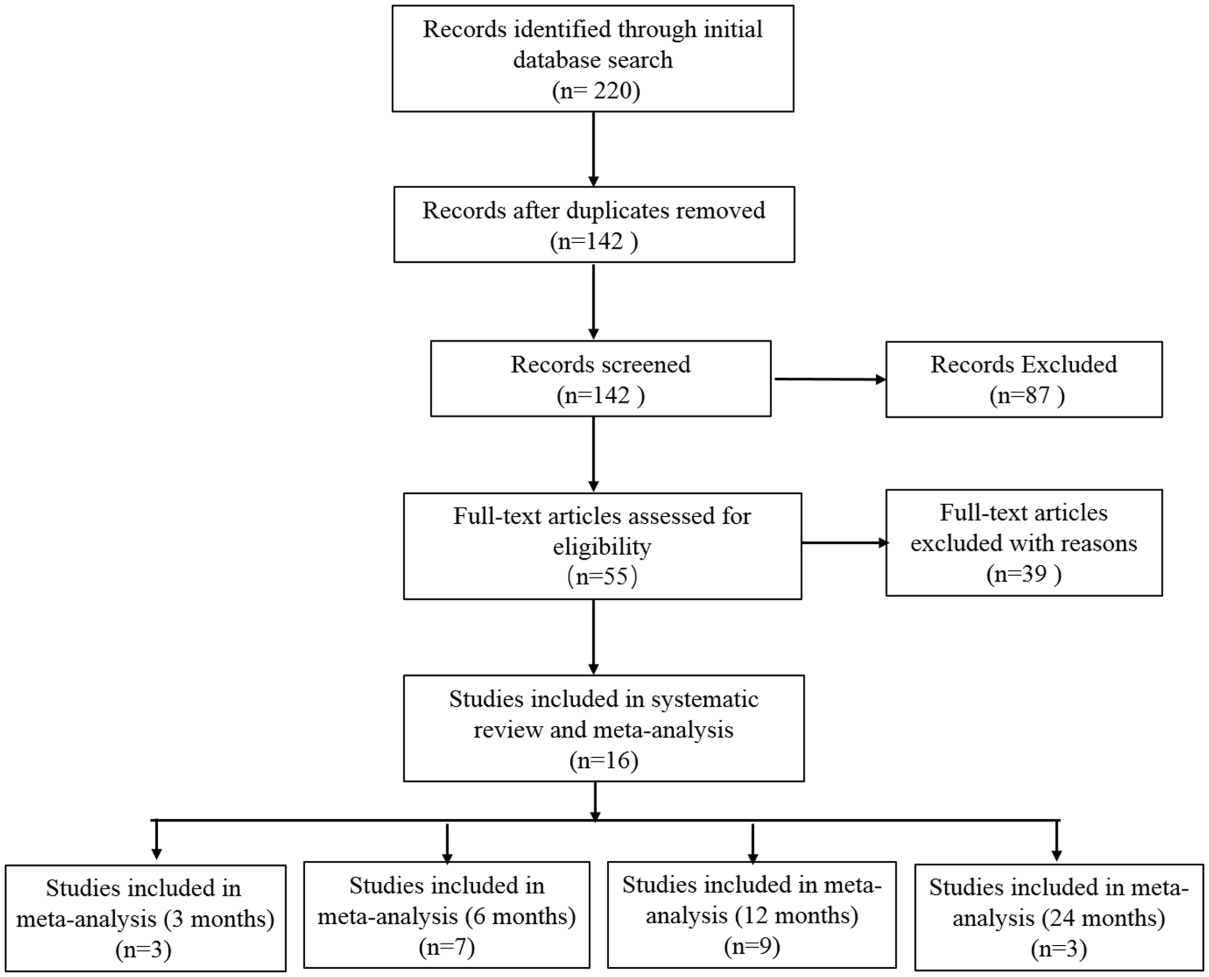
Flowchart of literature screening.

A total of 220 studies were identified through a comprehensive search of electronic databases and related bibliographies. The databases used for the search included Embase (150), Cochrane (7), PubMed (28) and CNKI (10). Based on the titles and abstracts, a total of 55 studies were deemed potentially eligible and selected for further evaluation. After reviewing the full texts, 16 studies were finally included according to the predetermined inclusion and exclusion criteria. The selection procedure is presented in Figure 1.

### 3.2 Study characteristics

In our review, a total of 16 studies were identified as prospective studies. Among these, 31.25% (5/16) studies were conducted in Asia, 25.00% (4/16) studies in Europe, 25.00% (4/16) studies in North America, and 18.75% (3/16) studies in South America. Journal of Biomedical Informatics (JBI) was used to evaluate the quality of the included references (Table 1). Meanwhile, a total of 16 studies were ultimately included based on the predetermined criteria for inclusion and exclusion. These studies encompassed a total of 197 patients with DS who underwent treatment with VNS (Table 2). Among the references cited, there were 3 studies with a follow-up time of 3 months, 7 studies with a follow-up time of 6 months, 8 studies with a follow-up time of 12 months, 2 studies with a follow-up time of 18 months, 3 studies with a follow-up time of 24 months, and 2 studies with a follow-up time of 36 months (Table 3). Characteristics of the studies and participants are synthetized in Tables 2, 3.

**Table 2:** Characteristics of studies.

	Study	Year	Follow-up time	Country	The number of DS patients treated with VNS ≥ 50% reduction of seizure frequency	The number of DS patients treated with VNS	Further patient outcome	Study limitation	References
1	Nelia Zamponi	2011	12 months	Italy	4	8	Cognitive level was unchanged in all patients. 7 patients showed a slight improvement in alertness and communicative skills	Retrospective	(16)
2	Ricardo O. Cersósimo (1)	2011	≥24 months	Argentina	2	3	EEG abnormalities improved in the two responders	Retrospective	(17)
3	Ricardo O. Cersósimo (2)	2011	≥24 months	Argentina	2	3	Not reported	Retrospective	(18)
4	Servicio de Neurología	2011	12 months	Argentina	2	3	EEG abnormalities had improved in the two patients	Retrospective	(19)
5	Iren Orosz	2014	24 months	European	5	13	Not reported	Retrospective	(20)
6	Anastasia Dressler	2015	3 months	Austria	3	8	Not reported	Retrospective	(21)
7	Deepa Sirsi	2015	≥12 months	USA	3	8	2 patients were reported subjective improvement in alertness and interaction	Retrospective	(12)
8	Brian J. Dlouhy	2016	≥12 months	USA	4	6	Not reported	Retrospective	(22)
9	Stephen P. Fulton	2017	≥6 months	USA	9	12	Cognitive and speech improvements in 4 out of 9 responders	Retrospective	(11)
10	Rushna Ali	2017	unclear	USA	14	49	Not reported	Retrospective	(23)
11	Yijie Li	2019	≥6 months	China	2	3	Not reported	Retrospective	(24)
12	Tong Zhang	2020	≥3 months	China	3	4	Not reported	Retrospective	(25)
13	ZhiJi Wang	2020	≥6 months	China	10	20	Not reported	Retrospective	(26)
14	Song Ee Youn	2021	≥3 months	Republic of Korea	12	19	12 patients were reported a subjective improvement in cognition, communication skills, and general condition	Retrospective	(6)
15	Cem Boluk	2022	≥6 months	Turkey	8	10	Two patients of VNS therapy was discontinued due to adverse events	Retrospective	(12)
16	Han Xie	2022	12 months	China	3	4	Not reported	Retrospective	(10)

**Table 3:** Characteristics of studies in the different follow-up time.

	Study	Year	Country	The number of DS patients treated with VNS ≥ 50% reduction of seizure frequency	The number of DS patients treated with VNS	References
**Follow-up time, 3 months**						
1	Anastasia Dressler	2014	Austria	3	8	(21)
2	Tong Zhang	2020	China	0	4	(25)
3	Song Ee Youn	2021	Republic of Korea	7	22	(6)
**Follow-up time, 6 months**						
1	Ricardo O. Cersósimo (1)	2011	Argentina	2	3	(17)
2	Iren Orosz	2014	European	2	16	(20)
3	Stephen P. Fulton	2017	USA	9	12	(11)
4	Yijie Li	2019	China	2	3	(24)
5	Tong Zhang	2020	China	2	4	(25)
6	Cem Boluk	2021	Turkey	6	10	(12)
7	Song Ee Youn	2021	Republic of Korea	8	22	(6)
**Follow-up time, 12 months**						
1	Servicio de Neurología	2011	Argentina	2	3	(19)
2	Nelia Zamponi	2011	Italy	4	8	(16)
3	Iren Orosz	2014	European	5	20	(20)
4	Deepa Sirsi	2015	USA	3	8	(12)
5	Brian J. Dlouhy	2016	USA	4	6	(22)
6	Tong Zhang	2020	China	3	4	(25)
7	Cem Boluk	2021	Turkey	8	10	(12)
8	Song Ee Youn	2021	Republic of Korea	8	22	(6)
9	Han Xie	2022	China	3	4	(10)
**Follow-up time, 18 months**						
1	Cem Boluk	2021	Turkey	8	10	(12)
2	Song Ee Youn	2021	Republic of Korea	9	22	(6)
**Follow-up time, 24 months**						
1	Iren Orosz	2014	European	5	13	(20)
2	Yijie Li	2019	China	2	3	(24)
3	Song Ee Youn	2021	Republic of Korea	12	22	(6)
**Follow-up time, 36 months**						
1	Ricardo O. Cersósimo (2)	2011	Argentina	2	3	(18)
2	Song Ee Youn	2021	Republic of Korea	12	19	(6)

### 3.3 Results of meta-analysis

**Figure 2:**
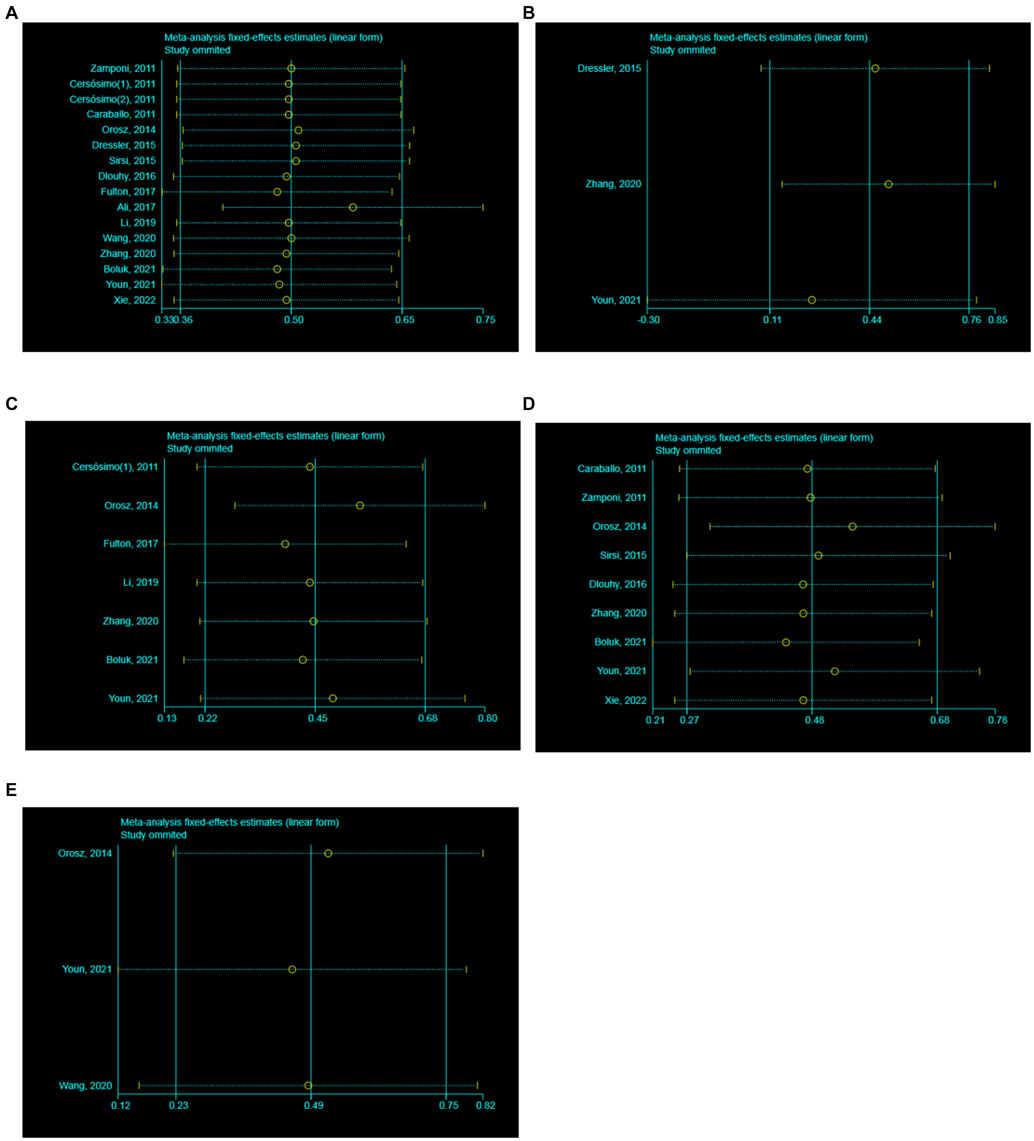
Sensitivity analysis. **(A)** All studies (the follow-up time is not considered). **(B)** The follow-up time of 3 months. **(C)** The follow-up time of 6 months. **(D)** The follow-up time of 12 months. **(E)** The follow-up time of 24 months.

The heterogeneity test of 16 literatures included in this study showed that *I*^2^ = 30.56% < 50%, and *p* = 0.12 > 0.1 in *Q* test. It is suggested that there is little heterogeneity among the literature selected in this study. Meanwhile, the meta-analysis was conducted to respectively evaluate the efficacy of VNS in the treatment of DS at 3, 6, 12 and 24 months. In the 3 studies with a follow-up time of 3 months, the results showed that *I*^2^ = 63.18% > 50%, and *p* = 0.07 < 0.1 in *Q* test. It is suggested that there is heterogeneity among the literature selected in these studies with a follow-up time of 3 months, reaching moderate heterogeneity. In the 7 studies with a follow-up time of 6 months, the results showed that *I*^2^ = 58.30% > 50%, and *p* = 0.03 < 0.1 in *Q* test. It is suggested that there is heterogeneity among the literature selected in these studies with a follow-up time of 6 months, reaching moderate heterogeneity. In the 9 studies with a follow-up time of 12 months, the results showed that *I*^2^ = 37.00% < 50%, and *p* = 0.12 > 0.1 in *Q* test. It is suggested that there is little heterogeneity among the literature selected in this study. In the 3 studies with a follow-up time of 24 months, the results showed that *I*^2^ = 0.00% < 50%, and *p* = 0.67 > 0.1 in *Q* test. It is suggested that there is little heterogeneity among the literature selected in this study. Consequently, further sensitivity analysis should be continued to investigate the causes of heterogeneity. A total of 16 literatures were subjected to sensitivity analysis in this study (Figures 2A–E). We found the reason for the significant heterogeneity in the studies with a follow-up time of 6 months was the inclusion of Orosz’s article the six articles. We recalculated after removing the Orosz’s article and found that *I*^2^ = 0.92% < 50%, and *p* = 0.42 > 0.1 in *Q* test. This suggests that there is little heterogeneity in the study. In addition, the 3 studies with a follow-up time of 3 months caused no significant interference with the results of this meta-analysis, which means that this study has good stability.

**Figure 3:**
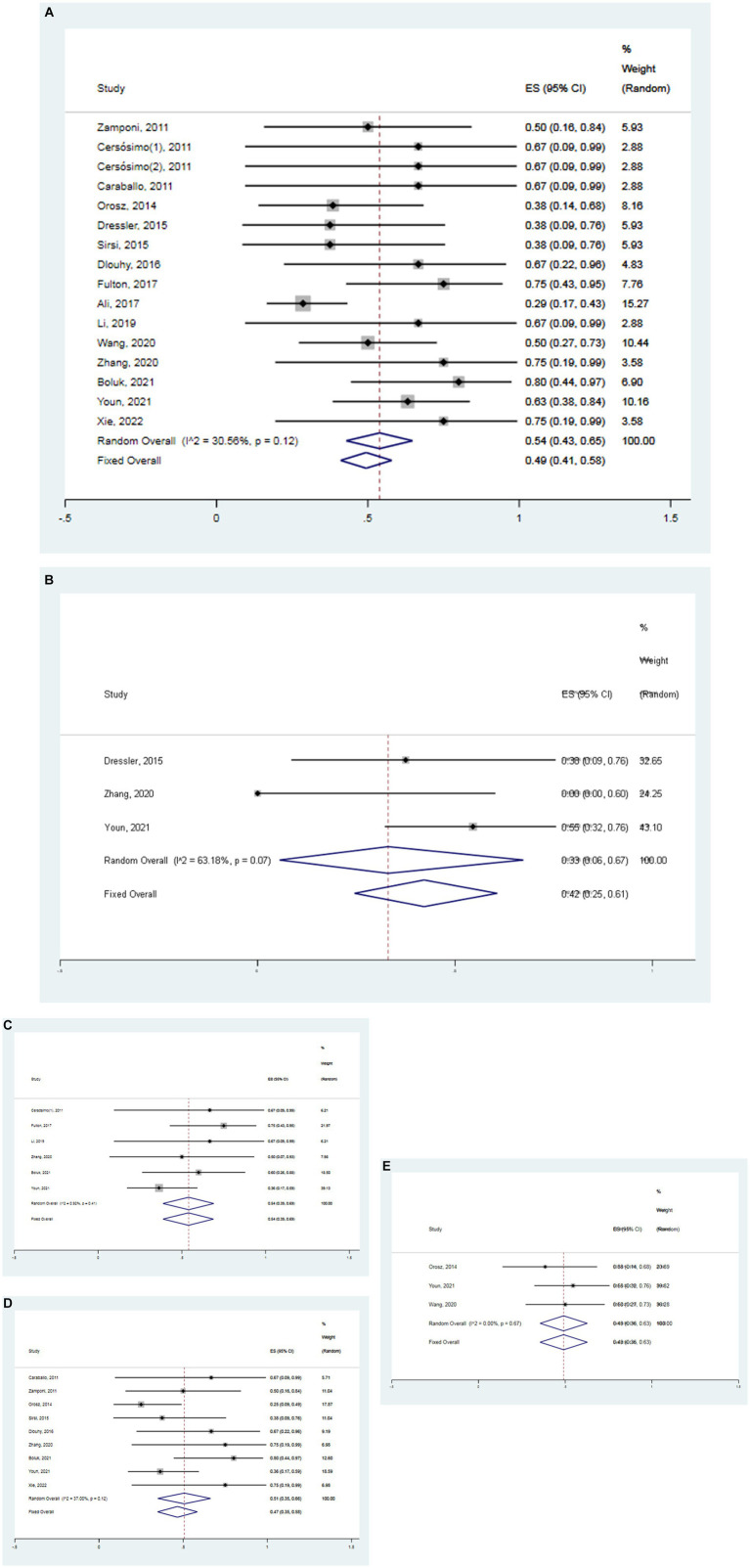
Forest map. **(A)** All studies (the follow-up time is not considered). **(B)** The follow-up time of 3 months. **(C)** The follow-up time of 6 months. **(D)** The follow-up time of 12 months. **(E)** The follow-up time of 24 months.

A meta-analysis based on random effects revealed that the overall effect size of 16 studies was 0.54, and the 95% confidence interval (CI) was 0.43–0.65, which was statistically significant (*z* = 12.81, *p* < 0.05) (Figure 3A). The finding suggests that VNS was significantly effective in the treatment of DS with an effectiveness rate of 54%. Additionally, separate meta-analyses were also conducted to respectively assess the effectiveness of VNS at 3, 6, 12 and 24 months in the treatment of DS. Meanwhile, the overall effect size of 3 studies was 0.42 (95% CI 0.25–0.61) in the patients with DS treated with VNS after 3 months, which was statistically significant (*z* = 2.89, *p* < 0.05) (Figure 3B). The overall effect size of 6 studies was 0.54 (95% CI 0.39–0.69) in the patients with DS treated with VNS after 6 months, which was statistically significant (*z* = 5.82, *p* < 0.05) (Figure 3C). The overall effect size of 9 studies was 0.51 (95% CI 0.39–0.66) in the patients with DS treated with VNS after 12 months which was statistically significant (*z* = 8.52, *p* < 0.05) (Figure 3D). The overall effect size of 3 studies was 0.49 (95% CI 0.36–0.63) in the patients with DS treated with VNS after 24 months which was statistically significant (*z* = 9.91, *p* < 0.05) (Figure 3E). It suggested that the effectiveness of VNS for DS respectively was 42, 54, 51 and 49% after the follow-up time of 3, 6, 12 and 24 months. In a word, VNS is effective in the treatment of DS. Within 6 months of treatment, the effectiveness of VNS increases significantly with the duration of treatment. In addition, the efficacy of this therapy can be concluded only up to 6 months.

**Figure 4:**
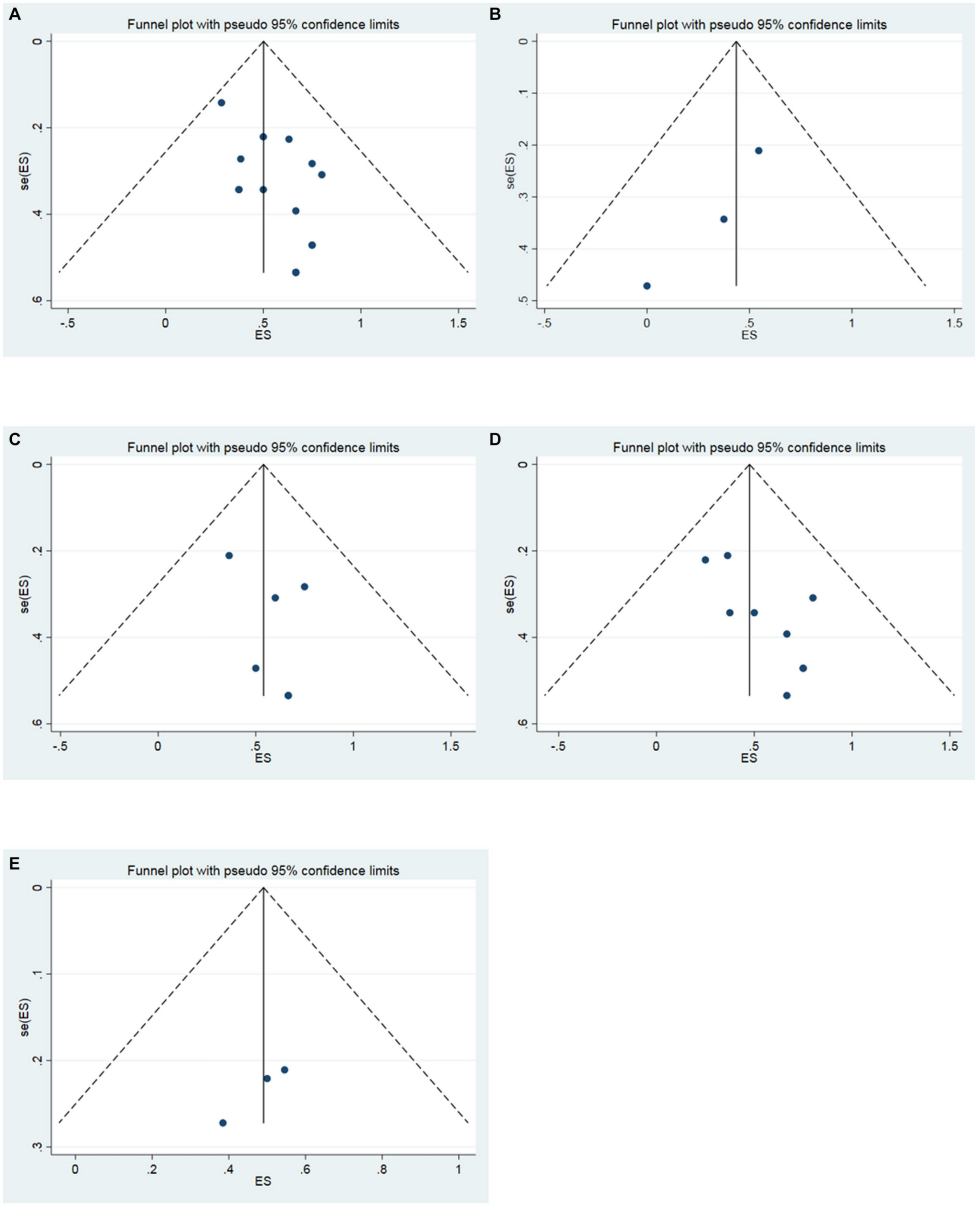
Funnel plot. **(A)** All studies (the follow-up time is not considered). **(B)** The follow-up time of 3 months. **(C)** The follow-up time of 6 months. **(D)** The follow-up time of 12 months. **(E)** The follow-up time of 24 months.

The funnel plot was drawn to detect the existence of publication bias in this study. The funnel plot of 16 studies was symmetric, which meant that there was no publication bias (Figure 4A). In addition, the funnel plots were also performed to respectively evaluate the publication bias in the studies of the follow-up time of 3, 6, and 12 months. In the follow-up time of 3 months, the funnel plot of 3 studies was symmetric (Figure 4B). In the follow-up time of 6 months, the funnel plot of 6 studies was symmetric (Figure 4C). In the follow-up time of 12 months, the funnel plot of 9 studies was symmetric (Figure 4D). In the follow-up time of 24 months, the funnel plot of 3 studies was symmetric (Figure 4E). Three symmetric funnel plots suggested that there was no publication bias in the studies in the follow-up time of 3, 6, 12 and 24 months.

## 4 Discussion

DS is a refractory developmental and epileptic encephalopathy characterized by seizures, developmental delay, and cognitive impairment, with a variety of comorbidities including autism-like behavior, speech dysfunction, and ataxia (1, 2). VNS has been found to be an effective treatment for DRE and can also be used in the treatment of DS (9). This meta-analysis reveals that VNS is significantly effective in treating DS with an overall effective rate of 54%. Furthermore, the effective rate of VNS for DS respectively was found to be 42, 54, 51 and 49% after 3, 6, 12 and 24 months, respectively. These findings suggest that VNS is significantly effective in the treatment of DS and it could be an effective treatment option for DS. It is worth noting that the effectiveness of VNS in treating DS significantly increased within first 6 months of treatment. However, it is important to mention that the efficacy of this therapy can be concluded only up to 6 months.

In epilepsy, DRE affects approximately one-third of patients and is associated with cognitive impairment and a decreased quality of life (27). In children, the epileptogenesis can be caused by various factors, and there has been significant interest in the treatment of DREs, including DS, has attracted much attention (9). DS is known to be a rare, severe, and refractory developmental and epileptic encephalopathy, with over 90% of DS patients who having a pathogenic mutation in *SCN1A* gene (29). Clinically, the main manifestations of DS are intractable seizures, developmental impairments, and movement abnormalities. Despite the availability of multiple ASMs, seizures in the DS patients remain poorly controlled with nearly half of DS patients still experiencing at least three tonic-clonic seizures per month (29). Therefore, it is essential to explore effective treatment approaches for DS.

VNS is an established, effective treatment for medically refractory epilepsy (MRE) (30). VNS may improve seizure control, but its role in children with DRE is not well described. A meta-analysis of 480 patients with LGS found that 54% had more than a 50% reduction in seizure frequency after treatment with VNS (31). In a meta-analysis about DRE, VNS was able to significantly reduce seizures and improve quality of life in patients with TSC, while there was less reduction in seizures in patients with DS (9). Meanwhile, a previous meta-analysis has also found that adjunctive VNS elicits a 50% reduction in seizure frequency in 55.9% of patients with DS with a median reduction of 55% in seizure frequency (15). Thereby, the long-term follow-up studies are necessary to explore the response of DS to VNS in these children. In our study, this meta-analysis of 16 articles comprising 178 patients with DS found that 52% of patients experienced more than 50% seizure frequency reduction after treatment with VNS. The total effect size of 16 studies was 0.54 (95% CI 0.43–0.65), which was statistically significant. This study suggests that VNS is effective in the treatment of DS and it could be an effective treatment option for DS. However, among the 16 included studies, there are one study where the follow-up time is not clear (23).

Our study was also further respectively analyzed the effectiveness of VNS in the DS patients with the follow-up time of 3, 6, 12 and 24 months. The results showed that the effective rate of VNS for DS respectively was 42, 54, 51 and 49% after the follow-up time of 3, 6, 12 and 24 months. Among these references, there were 3 studies with a follow-up time of 3 months, 6 studies with a follow-up time of 6 months, 9 studies with a follow-up time of 12 months, 2 studies with a follow-up time of 18 months, 3 studies with a follow-up time of 24 months and 2 studies with a follow-up time of 36 months. Meanwhile, the total effect size was respectively 0.42 (95% CI 0.25–0.61), 0.54 (95% CI 0.39–0.69), 0.51 (95% CI 0.39–0.66) and 0.49 (95% CI 0.36–0.63) in the patients with DS treated with VNS after 3, 6, 12 and 24 months, which was statistically significant (*p* < 0.05). These results suggest that VNS is effective in the treatment of DS and it could be an effective treatment option for DS. This study further indicates that VNS is effective in the treatment of DS. Within 6 months of treatment for DS, the effectiveness of VNS increased significantly with the duration of treatment. In addition, the efficacy of this therapy can be concluded only up to 6 months. Thus, VNS is an effective treatment option for DS patients, when seizures are difficult to control.

However, there were some limitations to our study. Firstly, the efficacy and safety of VNS is based on short-term treatment. In the 3 studies with a follow-up time of 3 months, and 7 studies with a follow-up time of 6 months, the results showed the significant heterogeneity (*I*^2^ > 50%, *p* < 0.1). However, we found the reason for the significant heterogeneity in the studies with a follow-up time of 6 months was the inclusion of Orosz’s article the six articles through sensitivity analysis. We calculated again after removing the Orosz’s article and found that *I*^2^ = 0.92% < 50%, and *p* = 0.42 > 0.1 in *Q* test, which suggested that there is no heterogeneity. In addition, sensitivity analysis also showed that 3 studies with a follow-up time of 3 months caused no great interference to the results of this meta-analysis, which means that this study has good stability. This meta-analysis does not allow us to reach a definitive conclusion regarding the effectiveness of VNS after 18 and 36 months in the treatment of DS. Thus, the long-term efficacy of VNS for DS still needs to evaluated. Secondly, only two trials were included in the current meta-analysis with a follow-up time of 18 or 36 months, which lowers the applicability of this study, and the results should be cautiously interpreted. Lastly, without comprehensive data regarding age and *SCN1A* mutations, it is not possible to conduct subgroup analyses of DS.

Additionally, with regards to the factors that influence the effectiveness of VNS, some studies propose that a longer the duration of epilepsy does not necessarily indicate a worse efficacy of VNS treatment (28, 32). However, there are also studies that showed that the shorter the duration of epilepsy in patients at the time of VNS implantation, often indicating that the better prognosis (33–36). The reasons may be as follows: (1) After a period of VNS treatment, the neural network of children with epilepsy will have benign lasting changes, and the earlier this change occurs, the better the effect (32). (2) Prolonged seizures can cause irreversible damage to the central nervous system, so early intervention will produce better results (33). Therefore, it is imperative to utilize VNS as an early intervention in the clinical management of DS.

## 5 Conclusion

This study suggests that VNS is effective in the treatment of DS and it could be considered as a viable treatment option for DS. Meanwhile, within 6 months of treatment for DS, the effectiveness of VNS increased significantly with the duration of treatment. In addition, the efficacy of this therapy can be concluded only up to 6 months. However, few studies have focused on VNS for DS, and there is a lack of high-quality evidence. Moreover, it is crucial to conduct high-quality randomized controlled trials are needed to confirm the effectiveness of VNS in treating DS.

## Data availability statement

The original contributions presented in the study are included in the article/supplementary material, further inquiries can be directed to the corresponding authors.

## Author contributions

SC: Conceptualization, Data curation, Formal analysis, Investigation, Methodology, Project administration, Writing – original draft, Writing – review & editing. ML: Conceptualization, Data curation, Formal analysis, Funding acquisition, Investigation, Methodology, Project administration, Resources, Software, Supervision, Validation, Visualization, Writing – review & editing. MH: Conceptualization, Data curation, Formal analysis, Funding acquisition, Investigation, Methodology, Project administration, Writing – review & editing.
